# Identification of Immune-Related Prognostic Biomarkers Associated with HPV-Positive Head and Neck Squamous Cell Carcinoma

**DOI:** 10.1155/2021/6661625

**Published:** 2021-01-08

**Authors:** Yifei Chen, Jin Nie, Xiangsheng Li, Tao Fan, Xiaowen Deng, Dan Liang, Guilin Song

**Affiliations:** Department of Otolaryngology Head and Neck Surgery, Affiliated Changsha Hospital of Hunan Normal University, 70 Lushan Road, Changsha, Hunan 410013, China

## Abstract

**Background:**

As a type of malignant tumor, head and neck squamous cell carcinoma (HNSCC) seriously threatens human health. This study is aimed at constructing a new, reliable prognostic model.

**Method:**

The gene expression profile data of HNSCC patients were downloaded from the Gene Expression Omnibus and The Cancer Genome Atlas databases. The immune-related differentially expressed genes (IRDEGs) related to HNSCC were identified. We then used Cox regression analysis and least absolute shrinkage and selection operator (LASSO) analysis to explore IRDEGs related to the HNSCC prognosis and to construct and validate a risk scoring model and used ESTIMATE to evaluate tumor immune infiltration in HNSCC patients. Finally, we validated IGSF5 expression and function in HNSCC cells.

**Results:**

A total of 1,195 IRDEGs were found from the GSE65858 dataset. Thirty-one of the 1,195 IRDEGs were associated with the prognosis of HNSCC. Nine key IRDEGs were further selected using the LASSO method, and a risk scoring model was established for predicting the survival of HNSCC patients. According to the risk scoring model, the prognosis of patients in the high-risk group was worse than that of the low-risk group; the high-risk group had significantly higher immune scores than the low-risk group; and between the high- and low-risk samples, there were significant differences in the proportion of 10 types of cells, including naive cells, plasma cells, and resting CD4^+^ memory T cells. IGSF5 has low expression in HNSCC, and overexpression of IGSF5 significantly impaired HNSCC cell proliferation.

**Conclusion:**

This prognostic risk assessment model can help systematically evaluate the survival prognosis of HNSCC patients and provides a new research direction for the improvement of the survival prognosis of HNSCC patients in clinical practice.

## 1. Introduction

Head and neck squamous cell carcinoma (HNSCC) is an epithelial tumor that develops in the mucous membranes of the mouth, oropharynx, larynx, or hypopharynx [1]. According to the “Global Cancer Statistics 2018,” it is estimated that there are more than 800,000 new HNSCC cases each year [2]. HNSCC is associated with several risk factors, including tobacco use, alcohol consumption, and betel nut chewing (*Areca catechu*) [3]. Despite the reduction in HNSCC cases caused by these risk factors, the number of HNSCC cases caused by human papillomavirus (HPV) is increasing, and the overall prevalence is gradually rising [4]. Recently, convincing results have clearly demonstrated that HNSCC patients with HPV positivity and HPV negativity are completely different in terms of molecular markers, clinical manifestations, and response to treatment. As the most common subtype of infection, HPV16 infection accounted for approximately 80% of the HPV infections in HNSCC patients [5]. Moreover, HNSCC patients who did not smoke or drink alcohol but had HPV infection were already in the middle and late stages when they were diagnosed. Approximately 40-60% of these patients experience relapse after comprehensive treatment, including surgery, radiotherapy, and chemotherapy, and they do not respond to subsequent treatment measures [6]. Therefore, it is crucial to evaluate the prognosis of HNSCC patients with HPV infection. Currently, the clinical evaluation of the prognosis of HNSCC patients is mainly done through traditional tumor, node, metastases (TNM) staging. The accuracy of this prediction method is limited, and the prognostic stratification of patients is not accurate. Therefore, to better guide the clinical improvement of the prognosis of HNSCC patients with HPV infection, it is imperative to explore a stable and efficient prognostic model.

Immune escape is an important factor affecting the occurrence and development of malignant tumors, including HNSCC [7]. Immune-related biomarkers can define not only the immune status of patients but also the biological behavior of HNSCC. Many studies have provided evidence for the role of immune cells in the development of HPV-induced HNSCC. For example, CD4^+^ T cell infiltration in HPV^+^ HNSCC patients was higher than that of HPV^–^ HNSCC patients [8]. The levels of cytokines and chemokines in HPV^+^ HNSCC patients were higher than those of HPV^–^ patients [9]. Some studies have already explored a model for assessing the survival prognosis of HNSCC using immune-related genes [10]. These studies have had some limitations, such as the failure to perform their analyses in terms of the immune and stromal cell components in the tumor microenvironment, and their descriptions of the molecular characteristics of the tumor-immune interaction have been unclear, especially regarding the prognostic evaluation of HNSCC. Many components of the immune system are closely related to the occurrence and development of HNSCC [8]. Immune scores and stromal scores are emerging methods that can be used to characterize the tumor microenvironment to determine the invasion and infiltration capabilities of this tumor and can play a role in prognostic assessment [11].

Based on the gene expression profile data of HNSCC patients with HPV infection in the Gene Expression Omnibus (GEO) database and incorporating the known immune-related genes (IRGs) in the InnateDB database and the ImmPort database, we selected novel immune biomarkers related to HPV infection using bioinformatic methods and established a prognostic evaluation model. This model can be used to assess survival prognosis and immune infiltration in HNSCC patients. This study will help improve the clinical diagnostic and prognostic evaluation of HNSCC patients and provide new targets for the treatment of HNSCC patients.

## 2. Materials and Methods

### 2.1. Data Possession

The gene expression profile data and clinical information of HNSCC patients in the GSE65858 dataset were obtained from the GEO database (https://www.ncbi.nlm.nih.gov/geo/). There were 60 cases of HPV16 infection and 196 cases of HPV negativity. On September 12, 2019, the gene expression profile data and survival information of patients (494 cases) with HNSCC in The Cancer Genome Atlas (TCGA) database were obtained from the University of California, Santa Cruz, Xena website (https://xenabrowser.net/datapages/).

### 2.2. Selection of Immune-Related Differentially Expressed Genes (DEGs)

We used the package limma of R software (Version 3.44.3) to analyze the gene expression profile data of HNSCC patients in the GSE65858 dataset and screened the results according to adjusted *p* value (*p*_adj._) < 0.05 to select DEGs in HPV16-infected samples vs. HPV-uninfected samples. On September 12, 2019, IRGs were downloaded from the InnateDB database (https://www.innatedb.ca/) and the ImmPort database (https://www.immport.org). The intersection of DEGs and IRGs made up the list of immune-related differentially expressed genes (IRDEGs) for HNSCC patients.

### 2.3. IRDEG Enrichment Analysis

The functional enrichment of IRDEGs was analyzed using the ClusterProfiler package of R (Version 3.16.1). The significantly enriched Gene Ontology (GO) terms were identified based on the GO functional enrichment analysis results (*p*_adj._ < 0.05). Significantly enriched biological pathways were identified by the Kyoto Encyclopedia of Genes and Genomes (KEGG) pathway enrichment analysis (*p*_adj._ < 0.05).

### 2.4. Weighted Gene Coexpression Network Analysis (WGCNA), Survival Analysis, and Gene Set Enrichment Analysis (GSEA)

WGCNA was performed on IRDEGs, in which the clinical information of patients and the expression levels of IRDEGs were all calculated by WGCNA. Base

d on the topological overlap measure model, genes were clustered using the average linkage hierarchical clustering method to obtain different gene modules. Module eigengenes were defined as the first principal component of each gene module. The Pearson correlation coefficient between the model eigengene of each module and the clinical information of the samples was calculated to obtain the gene significance (GS). The higher the GS was, the more important the module was, indicating how more correlated this module was to HPV16-infected samples. Then, the IRDEGs in the key modules derived from WGCNA were used for survival analysis using the Kaplan-Meier method, and the survival-related genes among IRDEGs were identified.

### 2.5. Establishment and Validation of a Prognostic Model

The survival-associated core IRDEGs were further selected from among the survival-related IRDEGs using the least absolute shrinkage and selection operator (LASSO) method. A risk scoring model that could predict the prognosis of the samples was constructed using these core IRDEGs. The risk score of each sample was then calculated based on the model. Based on the median risk score of all HNSCC patients, HNSCC patients were divided into high-risk and low-risk groups. Kaplan-Meier survival analysis was used to analyze the survival prognosis of patients in the high-risk and low-risk groups, and the diagnostic value of the model was analyzed using the receiver operating characteristic (ROC) curve and was validated on the data of HNSCC patients in the TCGA database. On this basis, we grouped all patients with HNSCC according to different clinical characteristics and used the Kaplan-Meier method to analyze the stability of the predictive ability of the prognostic model after grouping. Finally, we used univariate and multivariate Cox regression analyses to investigate the correlation between the prognostic model and other clinical characteristics (N stage, T stage, age, stage, and sex) to determine the independence of the model.

### 2.6. Immune Infiltration and Calculation of Immune Cell Components

We used the estimate package of R to calculate the immune score and stromal score of each HNSCC patient by inputting the gene expression profile of each HNSCC patient, combined with the specific gene expression characteristics of immunization and stromal cells. Based on the median risk score of all HNSCC patients, HNSCC patients were divided into high-risk and low-risk groups, and the differences in the immune score and stromal score between the two groups were analyzed. We further used CIBERSORT and the LM22 matrix (a signature matrix containing 22 functionally defined human immune subsets) to calculate the proportions of 22 phenotypes of human hematopoietic cells in each HNSCC patient. The sum of the proportions of all estimated immune cell types in each sample was equal to 1. Based on the median risk score of all HNSCC patients, HNSCC patients were divided into high-risk and low-risk groups, and the differences in the proportions of immune cells between the two groups were analyzed.

### 2.7. Cell Culture and Transfection

The human HNSCC cell lines Hep-2 and TU212 were gifts from the Cancer Research Institute of Central South University. These two cell lines were cultured in the RPMI-1640 medium supplemented with 10% FBS, in a humidified incubator at 37°C with 5% carbon dioxide. Lentiviruses including IGSF5 overexpression plasmid and vector for IGSF5 were obtained from GeneChem (Shanghai, China) for in vivo experiments. Cells were seeded at 60-70% confluence and infected with lentivirus culture (MOI is 10). The noninfected cells were eliminated by puromycin, and stable cell lines were selected with 4 *μ*g/ml puromycin treatment after 72 h of transfection. The efficiency of transfection was determined by Western blot.

### 2.8. Western Blot and Antibody

Antibodies against IGSF5 were from Thermo Fisher Scientific (catalog # PA5-80713, 1 : 1000, Thermo Fisher Scientific, MA, USA); GAPDH was from Proteintech (1 : 5000, Proteintech Group, Wuhan, China). The human HNSCC cell lines were lysed by the RIPA lysis buffer, which contains a protease inhibitor cocktail and phosphatase protease inhibitor cocktail. The BCA Protein Assay Kit (Beyotime Institute of Biotechnology, Shanghai, China) was used to detect the concentration of total protein. 10% SDS-PAGE was used to separate the total protein, and then, the protein was transferred to the PVDF membrane (Merck Millipore, USA). We block the membrane with 5% BSA (Sigma-Aldrich, Shanghai, China) for 1 hour. Then, the membrane was incubated with the antibody of IGSF5 at 4°C overnight; the membrane was washed with TBST six times. After incubation with secondary antibodies for 1 h at room temperature, the membrane was washed with TBST six times again. Finally, the bands were detected by the Odyssey CLx Infrared Imaging System (LI-COR Biosciences, NE, USA).

### 2.9. Cell Proliferation and Colony Formation Assays

We used the cell counting kit 8 (CCK8) assay (Dojindo, Kumamoto, Japan) to measure cell proliferation in 96-well plates. Cells were seeded at 4 × 10^3^ per well, with five replicates for each condition. CCK8 was added at 24, 48, 72, and 96 h and incubated at 37°C for 2 h. Cell numbers were determined by measuring the absorbance at 450 nm. We seeded 500 cells in each well of a 6-well plate in triplicate for each condition and incubated for 10 days. Then, the colonies were fixed by methanol and stained with crystal violet. The average colony counts were calculated. Each experiment was repeated three times.

### 2.10. Cell Apoptosis Analysis

Cells were harvested, washed twice with ice-cold phosphate-buffered solution (PBS), and fixed in 70% ice-cold ethanol, then incubated at 4°C overnight. Then, the cells were suspended following the manufacturer's protocol. Finally, a FACSCalibur flow cytometer was used for detection and FlowJo/ModFit LT software for data analysis (Becton Dickinson, NJ, USA). All experiments were performed three times.

## 3. Results

### 3.1. Selecting IRDEGs

**Figure 1:**
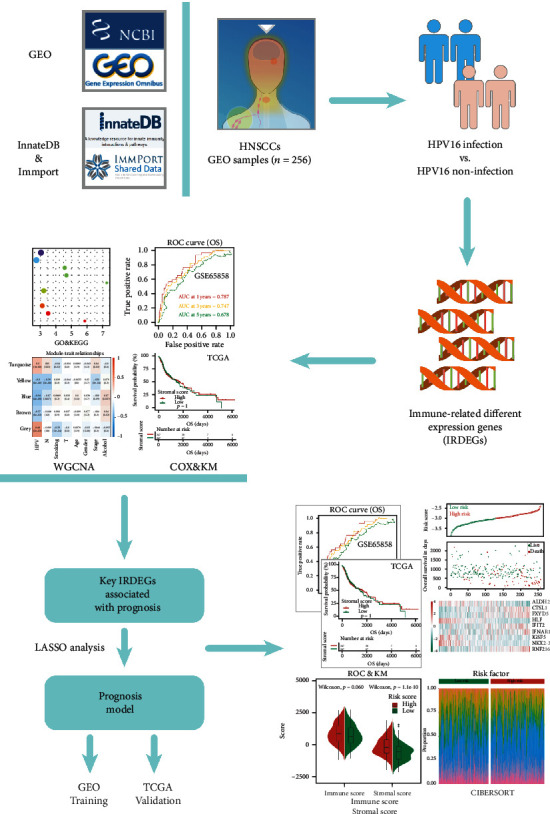
Flowchart of the present study.

**Figure 2:**
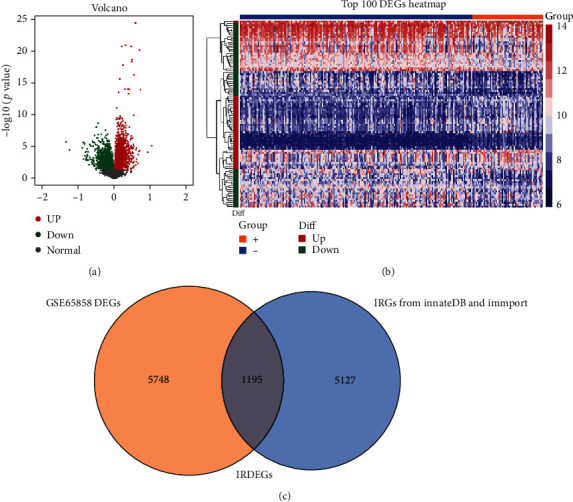
Overview of IRDEG profiling in HNSCC. (a) The DEGs between HNSCC tissues and normal tissues of dataset GSE65858. The DEGs identified in HNSCC were visualized in a volcano plot. The red and green points in the plot represent DEGs with statistical significance (*p*_adj._ < 0.05). (b) Heat map of the top 100 DEGs. (c) The two datasets had an overlap of 1,195 genes.

The design of this study design is illustrated, as shown in Figure 1. To clarify the effects of HPV16 infection on the immune system of HNSCC patients, we first downloaded the GSE65858 data from the GEO database (containing 60 HNSCC patients with HPV16 and 196 HNSCC patients without HPV infection) to screen for DEGs. A total of 5,748 DEGs were identified (*p* < 0.05), of which 3218 were upregulated and 2,530 were downregulated (Figures 2(a) and 2(b)). Then, a total of 5,127 IRGs were downloaded from the InnateDB database and the ImmPort database. The intersection of the IRGs and DEGs was a total of 1,195 genes, called IRDEGs associated with HPV16-related HNSCC (Figure 2(c)). These findings suggest that HPV infection could cause an immune response in HNSCC patients.

### 3.2. IRDEG Enrichment Analysis

**Figure 3:**
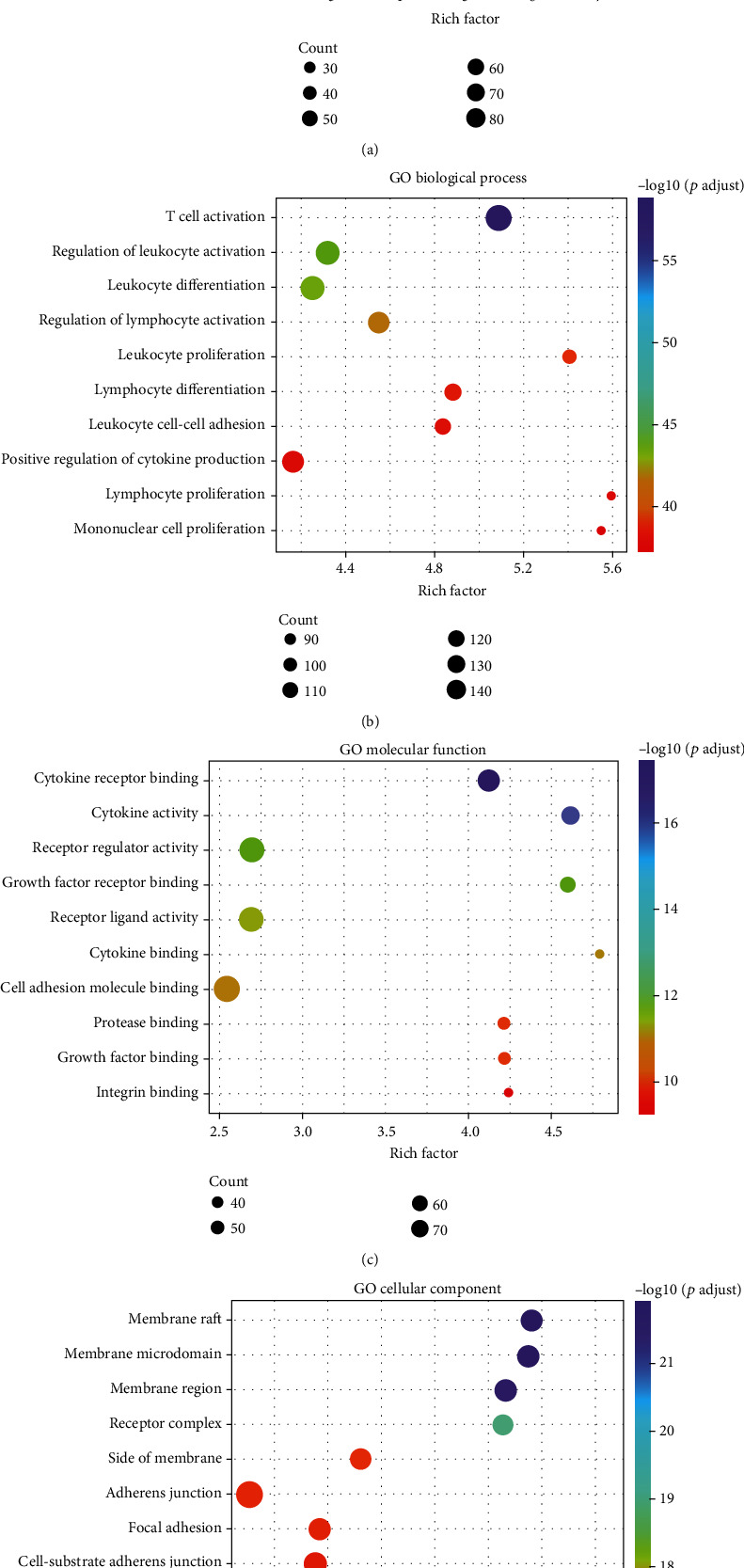
KEGG and GO analysis of the overlapping IRDEGs in HNSCC: (a) KEGG pathways; (b) biological processes; (c) molecular functions; (d) cellular components. Functional and pathway enrichment of IRDEGs is displayed in bubble charts.

Next, to further clarify the signaling pathways and molecular functions in which these IRDEGs were involved, functional enrichment analysis was performed on all 1,195 IRDEGs. The KEGG results showed that these genes played regulatory roles in important immune-related pathways, such as primary immunodeficiency, and in tumor-related signaling pathways, such as the PI3K-Akt signaling pathway (Figure 3(a)). Our group further used GO analysis to categorize the functions of these genes, and similar results were obtained: these genes not only participate in T cell activation, regulation of leukocyte activation, leukocyte differentiation, and other immune-related biological processes (Figure 3(b)) but also regulate cytokine receptor binding, cytokine activity, receptor regulator activity, and other tumor-related molecular functions (Figure 3(c)). Some encoded proteins that are important components of membrane rafts, membrane microdomains, and membrane regions (Figure 3(d)). These results further indicate that HPV infection indeed had a certain impact on the immune system of HNSCC patients, thus affecting tumor occurrence and development.

### 3.3. WGCNA and Survival Analysis

**Figure 4:**
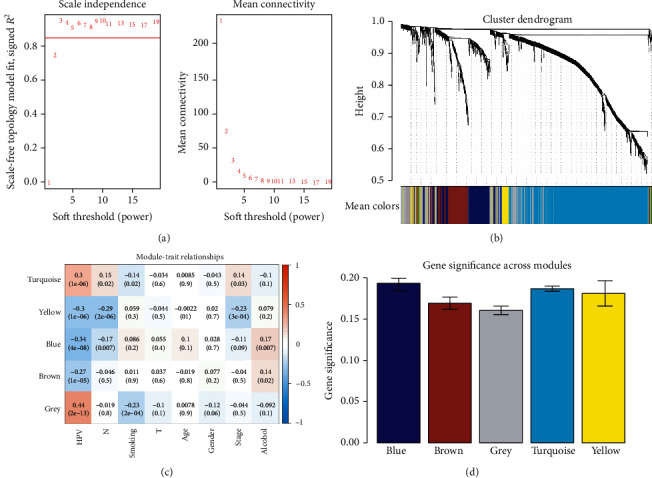
Weighted IRDEGs of the HNSCC expression network. (a) The scale-free fit index for soft-thresholding powers. The soft-thresholding power in the WGCNA was determined based on the scale-free *R*^2^ (*R*^2^ = 0.85). The left panel presents the relationship between the soft threshold and the scale-free *R*^2^. The right panel presents the relationship between the soft threshold and mean connectivity. (b) A dendrogram of the differentially expressed genes clustered based on different metrics. Each branch in the figure represents one gene, and the color below it represents one expression module. (c) Heatmap showing the correlation between the gene module and clinical traits. The turquoise module contained 596 IRGs, while the blue module contained 186 IRGs. The correlation coefficient in each cell represents the correlation between gene module and clinical traits, which decrease in magnitude from red to blue. The turquoise module showed the highest positive correlation with survival, while the blue module showed the highest negative correlation with survival. (d) Distribution of average GS and errors in the modules associated with the overall survival of HNSCC patients. Based on the average linkage hierarchical clustering and the soft-thresholding power, six modules were identified. To determine the significance of each module, GS was calculated to measure the correlations between genes and sample traits. GS was defined as the log_10_ conversion of the *p* value in the linear regression between gene expression and clinical data (GS = log_10_*p*). The turquoise and blue modules showed high correlations with the survival of HNSCC patients.

After preliminary validation of the presence of IRDEGs and their signaling pathways, we further constructed a weighted gene coexpression network based on the 1,195 IRDEGs to identify key IRGs to the survival prognosis of HNSCC patients. To ensure that the network was a scale-free network, we chose the optimal *R*^2^ of 0.85 (Figure 4(a)). We clustered these genes using the average linkage hierarchical clustering method. A total of five modules were obtained (Figure 4(b)). The number of genes in each module was statistically analyzed as shown in Supplement Table 1. As shown in Figure 4(c), the color changing from red to blue represents the correlation coefficient decreasing from high to low. We also calculated the GS value of each gene module (Figure 4(d)). A higher GS meant that the module was more correlated with samples with HPV infection. Based on the results of the above two methods of analyzing the correlation between module and phenotype, the modules most correlated with HPV infection are colored turquoise (positive) and blue (negative).

**Figure 5:**
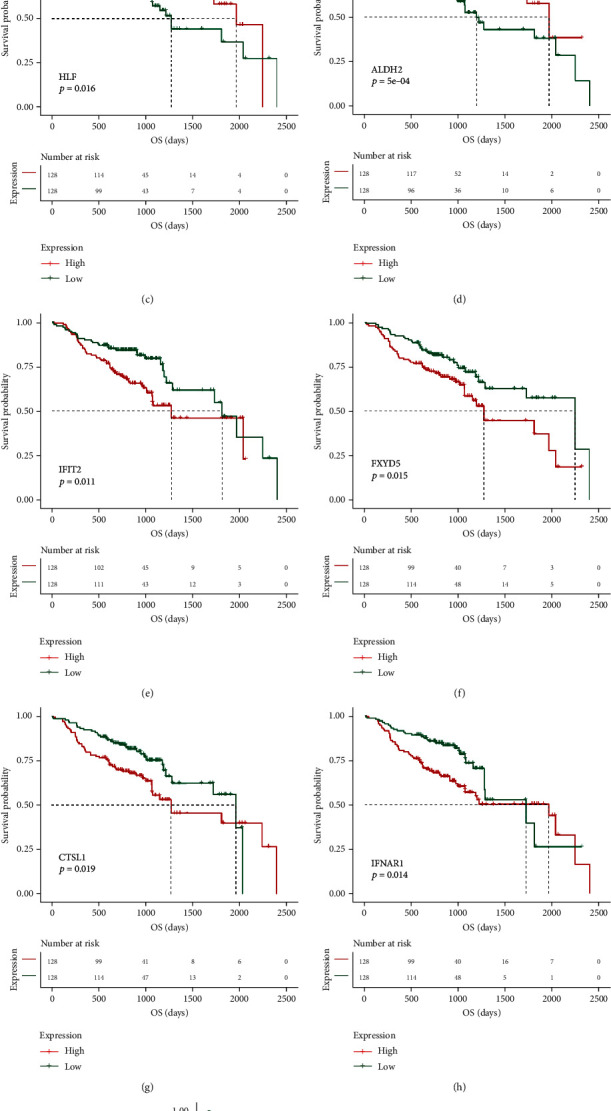
The prognostic value of the nine key IRDEGs ((a) *IGSF5*, (b) *NKX2-3*, (c) *HLF*, (d) *ALDH2*, (e) *IFIT2*, (f) *FXYD5*, (g) *CTSL1*, (h) *IFNAR1*, and (i) *RNF216*) in HNSCC patients in the overall survival curve.

We next performed univariate Cox survival analysis on the 782 key module genes (*N* = 596 + 186) included in the positive and negative correlation modules. A total of 31 IRDEGs that were correlated with survival prognosis were selected (the survival curves of some prognosis-related factors are shown in Figure 5, *p* < 0.05). In a word, high expression of IGSF5, NKX2-3, HLF, and ALDH2 showed a better overall survival rate compared with the low-expression group based on the Kaplan-Meier analysis. Low expression of IFIT2, FXYD5, CTSL1, IFNAR1, and RNF216 showed a poor overall survival rate compared with the high-expression group based on the Kaplan-Meier analysis.

### 3.4. Construction and Validation of Prognostic Models

**Figure 6:**
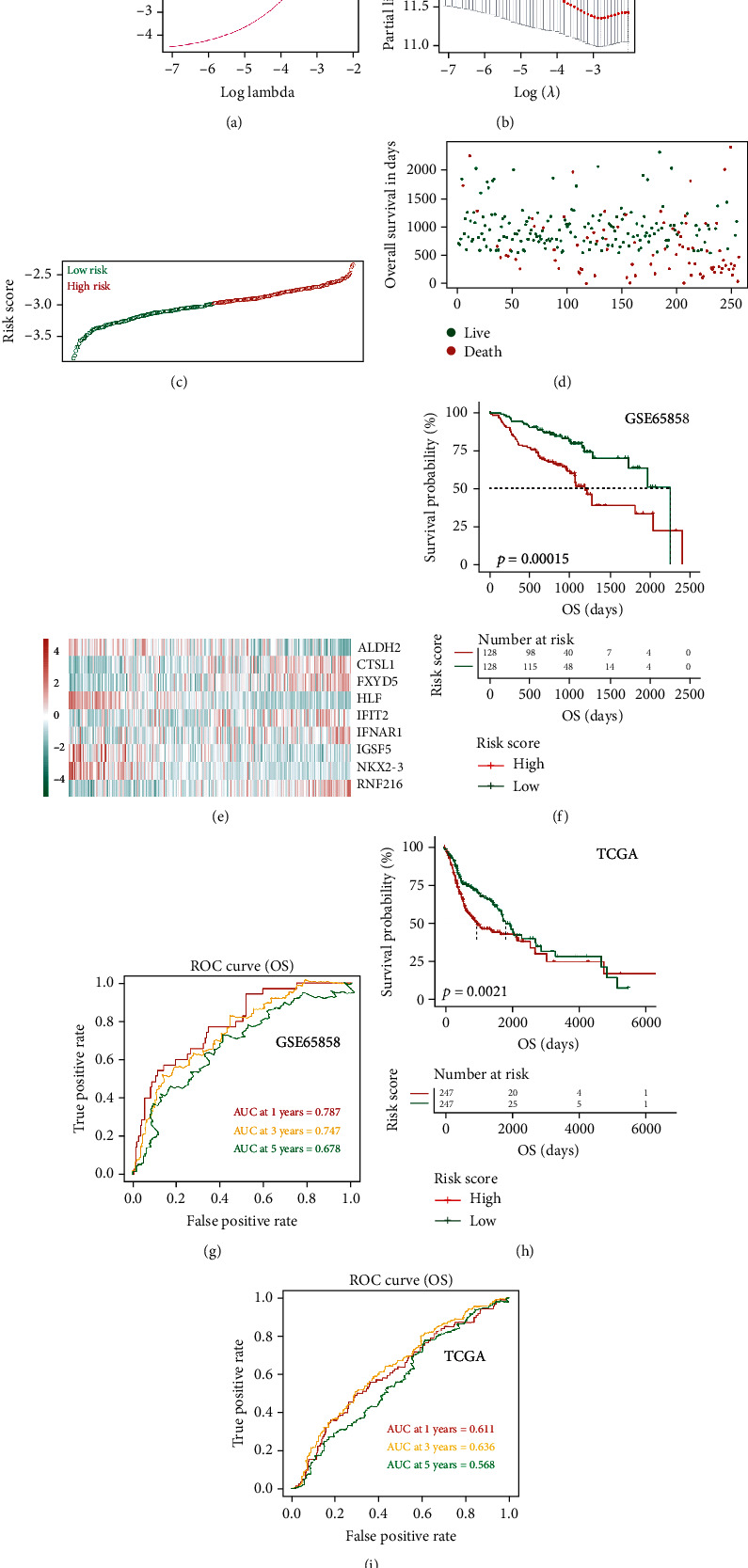
Construction of the IRDEG prognostic classifier, the distribution of time-dependent ROC curves, and Kaplan-Meier survival: (a, b) determination of the number of factors by LASSO analysis; (c) distribution of risk scores; (d) survival duration and status of patients; (e) heatmap of IRDEGs in the classifier; (f) Kaplan-Meier curve of the GSE65858 cohort; (g) ROC curve for the GSE65858 cohort; (h) Kaplan-Meier curve of the TCGA HNSCC cohort; (i) ROC curve for the TCGA HNSCC cohort.

To better evaluate the clinical prognosis of HNSCC patients through these IRDEGs, we selected nine key prognostic genes, including immunoglobulin superfamily member 5 (*IGSF5*), NK2 homeobox 3 (*NKX2-3*), hevein-like protein (*HLPF*), aldehyde dehydrogenase 2 family member (*ALDH2*), interferon-induced protein with tetratricopeptide repeats 2 (*IFIT2*), FXYD domain-containing ion transport regulator 5 (*FXYD5*), cathepsin L1 (*CTSL1*), interferon alpha and beta receptor subunit 1 (*IFNAR1*), and ring finger protein 216 (*RNF216*), using the LASSO method based on 31 survival-related IRDEGs (Figures 6(a)–6(i)). The expression levels of these nine key factors were weighted by the LASSO regression coefficients (Table 1), and the risk scoring model for predicting the survival prognosis of HNSCC patients was successfully established. The risk score of each sample was calculated based on the model. According to the median risk score of all HNSCC patients in GSE65858, HNSCC patients were divided into the high-risk and low-risk groups. There was a significant difference in prognosis between the two groups (Figure 6(f)). The prediction results of the model were evaluated using the ROC curve. The area under the ROC curve (AUC) for predicting the prognosis of HNSCC patients in the GNS65858 dataset at 1 year, 3 years, and 5 years reached 0.787, 0.747, and 0.678, respectively (Figure 6(g)), evidencing the effectiveness of this prognostic model. That is, this model could be used to evaluate the clinical prognosis of HNSCC patients. The above results were verified in HNSCC samples from the TCGA database to further confirm the effectiveness of the model in determining the prognosis of HNSCC patients (Figures 6(h) and 6(i)).

**Figure 7:**
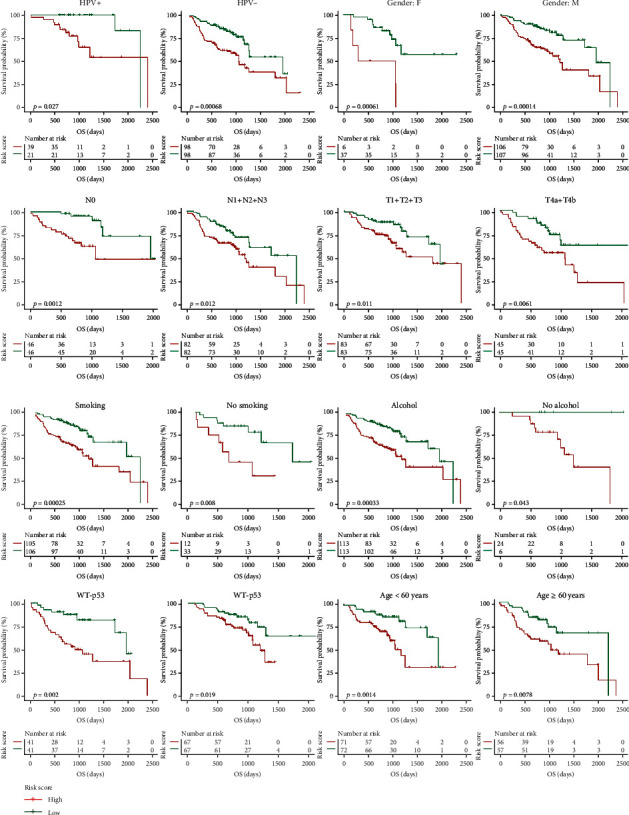
Effects of different clinical characteristics on the prognosis of HNSCC patients. Based on the risk score of 256 HNSCC patients in the GSE65858 dataset calculated by the prognostic evaluation model, patients were divided into high- and low-risk groups after first being stratified by clinical features such as HPV infection, sex, lymph node metastasis, degree of tumor infiltration, smoking, alcohol consumption, *TP53* gene mutation, and age. All these features had an impact on the prognosis of HNSCC patients.

In addition, to validate the stability of the model, 256 HNSCC samples in GSE65858 were grouped according to clinical characteristics. These HNSCC samples were divided into a high-risk group and a low-risk group based on the median model score. The results showed that whether we grouped according to HPV^+^, HPV^–^, female sex, male sex, N0, N1+N2+N3, T1+T2+T3, T4a+T4b, smoking, nonsmoking, alcohol, no alcohol, MT53, WT53, age ≥ 60, or age < 60, there was a significant difference in the prognosis between the high-risk group and the low-risk group (Figure 7). These findings further indicate that HPV infection is an important factor affecting the survival prognosis of HNSCC patients and that the predictive power of the predictive model is stable. Similarly, the stability of this prognostic model was also confirmed in HNSCC samples from TCGA (Supplement Figure 1).

Finally, to verify the independence of this prognostic evaluation model, we used univariate and multivariate Cox regression analyses to analyze the relationship between the prognostic evaluation model and other clinical factors in the experimental group. Univariate Cox regression analysis showed that the N stage, T stage, stage, age, and our prognostic model risk score were all significantly correlated with survival. Multivariate Cox regression analysis showed that the N stage, age, and prognostic model score were independent prognostic factors (Table 2). Applied to the TCGA validation set, univariate Cox regression analysis showed that age, sex, and prognostic model score were all significantly associated with survival; multivariate Cox regression analysis showed that age and the constructed prognostic model score were independent prognostic factors (Supplement Table 2). The above results indicate that the prognostic evaluation model based on IRDEGs is stable and reliable and can be used to evaluate the survival prognosis of HNSCC patients.

### 3.5. Immune Score and Stromal Score

**Figure 8:**
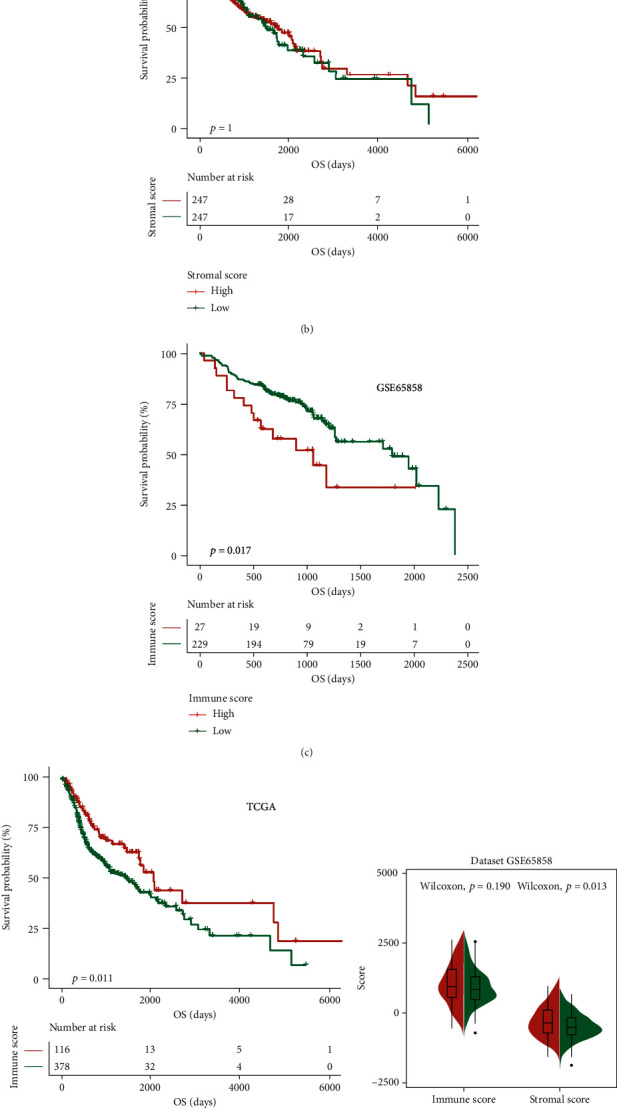
Effect of immune infiltration on the prognosis of HNSCC patients: (a, c) impact of the stromal score and immune score on overall survival in the GSE65858 cohort based on Kaplan-Meier analysis; (b, d) impact of the stromal score and immune score on overall survival in the TCGA HNSCC cohort based on Kaplan-Meier analysis; (e) association between immune score, stromal score, and risk score in the GSE65858 cohort; (f) association between immune score, stromal score, and risk score in the TCGA HNSCC cohort.

To further assess the relationship between immune infiltration and survival prognosis in HNSCC patients, we evaluated the immune score and stromal score. The HNSCC patients in the experimental group and the validation group were divided into two subgroups according to the median value of the immune score or the stromal score. There were significant differences in the prognosis between patients in the two subgroups of the experimental group (Figures 8(a) and 8(c), *p* < 0.05). There was no significant difference in the prognosis between the two subgroups divided according to the stromal score in the validation group (Figure 8(b), *p* > 0.05), but significant differences were identified between two subgroups divided according to the immune score (Figure 8(d), *p* < 0.05).

HNSCC patients in the experimental group and the validation group were divided into high-risk and low-risk groups according to the prognostic evaluation model. There was no significant difference in the immune score between patients in the high- and low-risk subgroups of the experimental group or the validation group (Figures 8(e) and 8(f), left part, *p* > 0.05), but there was a difference in the stromal score between the high- and low-risk patients of each group (Figures 8(e) and 8(f), right part, *p* < 0.05). These results suggest that the degree of immune infiltration affects the survival prognosis of HNSCC patients, and the prognostic evaluation model constructed by our research group could evaluate the degree of immune infiltration in HNSCC patients.

### 3.6. Immune Landscape of HNSCC Samples with Low/High Risk

**Figure 9:**
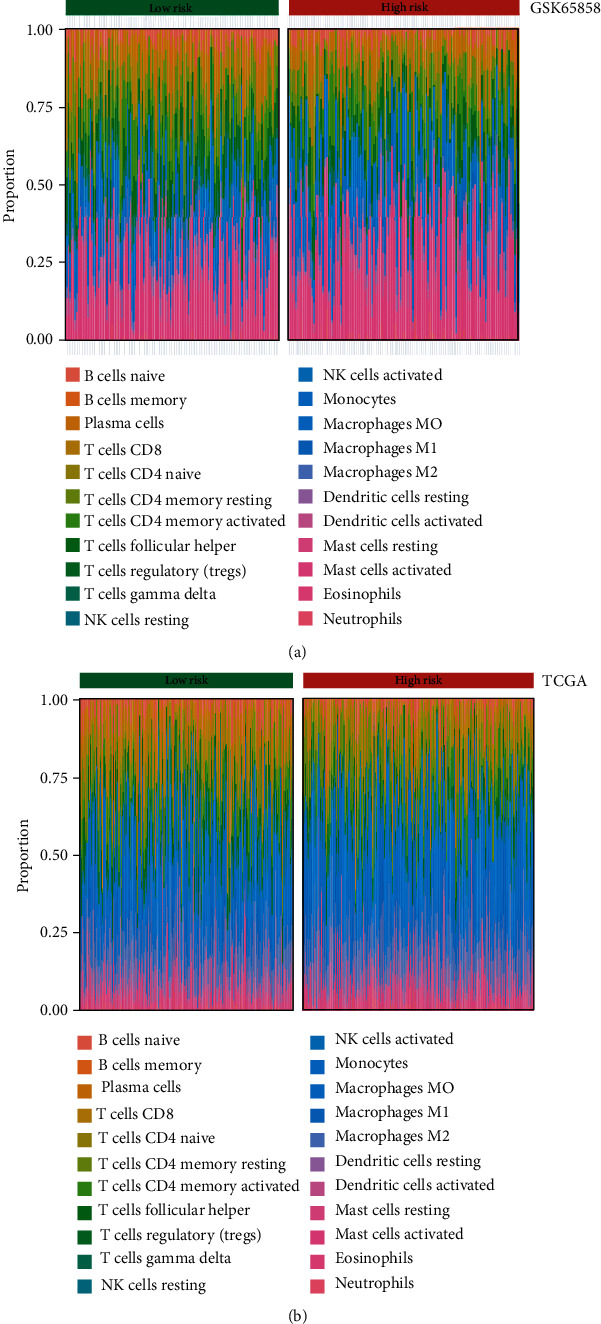
The proportions of immune cell types in HNSCC patients: (a) the mean proportions of 22 immune cell types in the GSE65858 cohort; (b) the mean proportions of 22 immune cell types in the TCGA HNSCC cohort.

After confirming the correlation between immune infiltration and the survival prognosis of HNSCC patients, we further explored the proportion of immune cells in the tumor tissues of HNSCC patients. The results showed that the proportion of immune cells in HNSCC samples differed between low- and high-risk HNSCC samples (Figure 9). Specifically, the analysis of the experimental group showed that the proportions of naive B cells, plasma cells, resting CD4^+^ memory T cells, follicular helper T cells, gamma delta T cells, M0 macrophages, resting dendritic cells, activated dendritic cells, activated mast cells, and neutrophils were significantly different between the two groups of samples (*p* < 0.05, Table 3). In the validation group, the proportion of 11 cell types, including naïve B cells, memory B cell, plasma cells, CD8^+^ T cells, resting CD4^+^ memory T cells, follicular helper T cells, regulatory T cells, gamma delta T cells, resting NK cells, M0 macrophages, and M2 macrophages, were significantly different between the two groups (Table 4).

## 4. Validation of IGSF5 Expression in HNSCC Cells from the Prognostic Model

**Figure 10:**
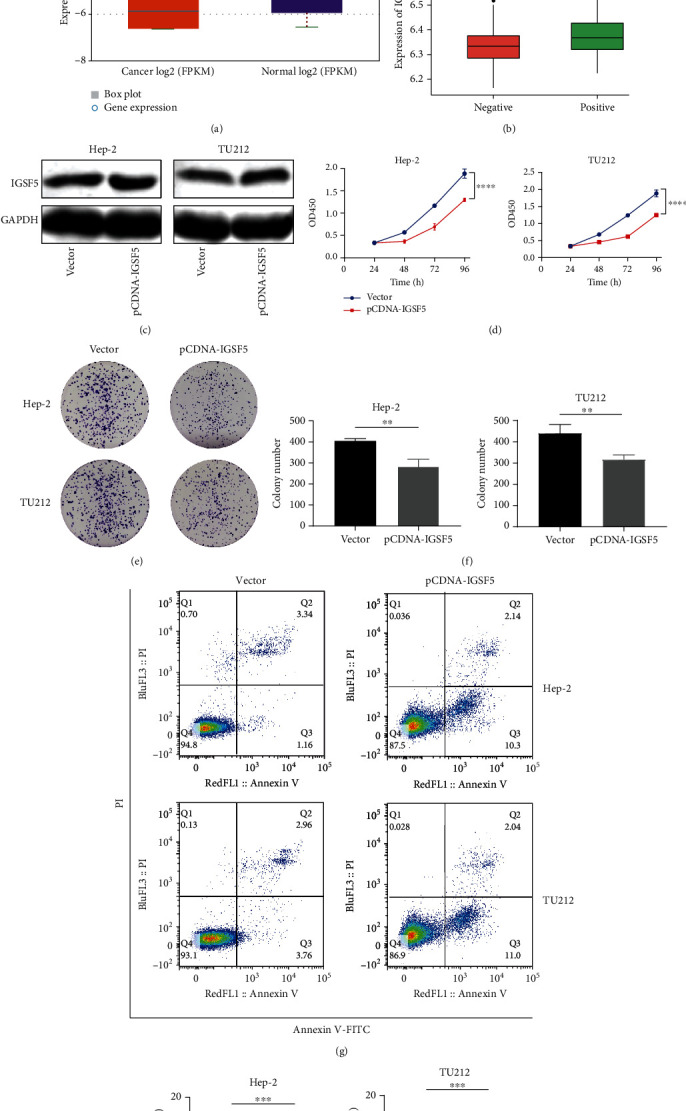
Overexpression of IGSF5 inhibits the proliferation of HNSCC cells. (a) IGSF5 had lower expression in HNSCC samples than in matched normal tissues from the TCGA database (normal = 44, tumor = 519). (b) IGSF5 in HPV-positive HNSCC tissues from the GSE65858 cohort was upregulated compared with that in HPV-negative tissues. (c) The expression of IGSF5 was detected by Western blot, and cells transfected with the IGSF5 overexpression plasmid differed significantly between the *IGSF5* overexpression (pCDNA-IGSF5) and control (vector) groups in Hep-2 and TU212 cells. GAPDH was used as an internal control. (d) Reduction in the proliferation ability of pCDNA-IGSF5 Hep-2 and TU212 cells compared with the control (vector) cells by the CCK8 assay. (e, f) Reduction in colony formation ability of pCDNA-IGSF5 Hep-2 and TU212 cells compared with the control (vector) cells by a colony formation assay. The bar graph indicates the number of colonies. (g, h) Cell apoptosis of pCDNA-IGSF5 Hep-2 and TU212 cells compared with the control (vector) was analyzed by flow cytometry. The rate of cell apoptosis is shown in the graphs. The results are presented as the mean ± s.d. and are representative of at least three independent experiments. ^∗^*p* < 0.05, ^∗∗^*p* < 0.01, ^∗∗∗^*p* < 0.001, and ^∗∗∗∗^*p* < 0.0001. ns: *p* > 0.05.

Since the IGSF5 gene is the most relevant factor in the model (LASSO coefficients = −0.68, Table 1), we selected it for preliminary verification. Firstly, we found that IGSF5 has low expression in HNSCC according to the data from starBase v3.0 (Figure 10(a)). And IGSF5 expression was significantly elevated in HNSCC patients infected with HPV (Figure 10(b)), which was consistent with the prognostic model. To validate the function of IGSF5, we upregulated the expression of IGSF5 in HNSCC cells (Figure 10(c)). Then, we found that overexpression of IGSF5 significantly impaired HNSCC cell proliferation and in Hep-2 and TU212 cells (Figures 10(d)–10(f)) and dramatically promotes cell apoptosis (Figures 10(g) and 10(h)).

## 5. Discussion

In recent years, tumor immunity and treatments related to it have been hot spots and challenges in HNSCC research. In this study, we first selected 31 key IRDEGs that affected the prognosis of HNSCC patients in terms of HNSCC patients with or without HPV16 infection and further used nine key IRDEGs to construct a stable and highly efficient prognostic evaluation model to evaluate the survival prognosis and immune infiltration of HNSCC patients in clinical practice. In our study, 5,127 IRGs were downloaded from the InnateDB database and ImmPort database, and 1,195 IRDEGs were obtained. In addition, KEGG and GO analysis confirmed that these genes not only are involved in many immunoregulatory pathways but also play important roles in tumor occurrence and development. She et al. previously studied the differences in IRGs among HNSCC patients with or without HPV infection. However, the IRGs in their study were limited to the 1,073 genes included in the ImmPort website. Moreover, their study did not examine the differential expression of IRGs between cancerous and paracancerous tissues of HNSCC patients [12]. Therefore, compared with our study, theirs has more limitations that greatly reduced the reliability of their results.

To evaluate the clinical prognosis of HNSCC patients, we built a prognostic model using the LASSO method. The nine core genes of the model, *IGSF5* [13], *NKX2-3* [14], *HLF* [15], *ALDH2* [16], *IFIT2* [17], *FXYD5* [18], *CTSL1* [19], *IFNAR1* [20], and *RNF216* [21], have been associated with tumor occurrence and development. *ALDH2* plays an important regulatory role in HNSCC [22] and the tumor immune response [23]. *HLF* is closely related to prognosis and drug resistance in HNSCC patients [24] and participates in the immune response of CD4^+^ and other immune cells [25]. *IFNAR1* is highly expressed in HNSCC patients, where it promotes the expression of programmed death-ligand 1 (PDL1) and programmed cell death protein 1 (PD1) in tumor cells [26]. These findings indirectly show that our risk prognosis model constructed based on the key IRDEGs related to prognosis was reliable. More importantly, the AUC values for predicting the survival of HNSCC patients in the GNS65858 dataset at 1 year, 3 years, and 5 years reached 0.787, 0.747, and 0.678, respectively, indicating that the prognostic evaluation results of the model were highly efficient. Our analysis results also demonstrated that there was a significant difference in survival prognosis in HNSCC patients with vs. without HPV infection. Sex, age, lymph node metastasis, tumor infiltration depth, smoking status, alcohol consumption, and *TP53* gene mutation were also closely correlated with the survival prognosis of HNSCC patients. Finally, we proved that IGSF5 was downregulated in HNSCC patients; overexpression of IGSF5 could inhibit HNSCC cell proliferation and induce cell apoptosis, which means that our predicted model is reliable.

In the tumor microenvironment, immune and stromal cells are the two main nontumor components. Information on immune cells and stromal cells in the tumor microenvironment can be used as an important indicator in evaluating the survival prognosis of cancer patients [27]. Therefore, based on the constructed risk prognostic model, we divided HNSCC patients into the high-risk group and the low-risk group and found that the two groups had significant differences not only in tumor infiltration depth and lymph node metastasis but also in the degree of immune infiltration in tumor tissue. The survival prognosis was even poorer in the group with higher immune and stromal scores. On this basis, we found that the immune cell components of patients in these two groups were also different, mainly in naïve B cells, memory B cells, plasma cells, CD8^+^ T cells, naive CD4^+^ T cells, resting CD4^+^ memory T cells, activated CD4^+^ memory T cells, and regulatory T cells, in line with previous findings [28]. For example, CD8^+^ T cells play an important role in the tumor microenvironment, which inhibits the proliferation and metastasis of tumor cells [29]. Our results indicate that CD8^+^ T cells were highly expressed in low immune risk groups. These findings further confirm the importance of including more IRGs in survival prognosis models.

In summary, this study constructed a more comprehensive immune-associated survival prognostic evaluation system for HNSCC patients. The core genes of this system, *IGSF5*, *NKX2-3*, *HLF*, *ALDH2*, *IFIT2*, *FXYD5*, *CTSL1*, *IFNAR1*, and *RNF216*, can be used as markers of HNSCC. This system can help predict survival, immune infiltration, and tumor metastasis in HNSCC patients. It provides an important reference for understanding HNSCC and finding new targets for diagnosis and treatment in clinical practice. In future studies, the expression and function of the nine key genes need further validation.

## 6. Conclusion

We constructed a prognostic risk assessment model to help systematically evaluate the survival prognosis of HNSCC patients and provide a new research direction for the improvement of the survival prognosis of HNSCC patients in clinical practice.

## Figures and Tables

**Table 1:** 9-gene immune signature.

Gene symbol	Univariate Cox regression analysis				LASSO coefficients
	HR	Low 95% CI	High 95% CI	*p* value	
IGSF5	1.65	1.07	2.54	0.023	-0.68
NKX2-3	1.58	1.02	2.43	0.039	-0.3
HLF	1.71	1.1	2.64	0.017	-0.26
ALDH2	2.18	1.39	3.43	0.001	-0.07
IFIT2	0.56	0.36	0.88	0.012	0.02
FXYD5	0.59	0.38	0.91	0.016	0.02
CTSL1	0.6	0.38	0.92	0.021	0.04
IFNAR1	0.58	0.37	0.9	0.015	0.13
RNF216	0.51	0.33	0.79	0.003	0.52

**Table 2:** Univariate and multivariate analyses of prognostic factors and overall survival of HNSCC patients in the GSE65858 cohort.

	Univariate analysis				Multivariate analysis			
	HR	Low 95% CI	High 95% CI	*p* value	HR	Low 95% CI	High 95% CI	*p* value
N	1.41	1.12	1.78	0.004	1.53	1.06	2.21	0.024
T	1.47	1.18	1.83	0.001	1.29	0.97	1.7	0.076
Age	1.03	1.01	1.05	0.006	1.03	1.01	1.05	0.015
Stage	1.56	1.16	2.09	0.003	0.88	0.53	1.45	0.614
Gender	1	0.57	1.75	0.999	—	—	—	—
Risk score	11.82	4.72	29.56	<0.001	8.84	3.67	21.26	<0.001

**Table 3:** Differential immune cell type expression was observed between the high and low RS groups in the GSE65858 cohort.

Cell type	Low risk	High risk	*p* value
Naive B cells	0.039804215	0.028293595	0.002849395
Memory B cells	0.003513616	0.004345782	0.691435185
Plasma cells	0.084088896	0.057558826	0.002982023
CD8 T cells	0.042427714	0.043767342	0.840225177
Naive CD4 T cells	0.012275351	0.012559372	0.938789785
Resting CD4 memory T cells	0.083830748	0.064374727	0.006572652
Activated CD4 memory T cells	0.080999535	0.081630012	0.948165785
Follicular helper T cells	0.087385368	0.074676429	0.019497779
Regulatory T cells (Tregs)	0.013609875	0.009772032	0.214696852
Gamma delta T cells	0.042151413	0.028734392	0.002338151
Resting NK cells	0.000999625	0.00106397	0.933127189
Activated NK cells	0.053364353	0.053284618	0.983751584
Monocytes	0.024330133	0.026670409	0.505003128
M0 macrophages	0.063840447	0.078844898	0.049219689
M1 macrophages	0.055566688	0.061100091	0.312529981
M2 macrophages	0.026315633	0.030067857	0.323696097
Resting dendritic cells	0.019759167	0.013892642	0.040243924
Activated dendritic cells	0.035258645	0.046148972	0.042403999
Resting mast cells	0.005683298	0.002321107	0.213976349
Activated mast cells	0.220380575	0.271912742	0.002425909
Eosinophils	0.000213091	0.000188235	0.914308033
Neutrophils	0.004201615	0.00879195	0.011608708

**Table 4:** Differential immune cell type expression was observed between the high- and low-RS groups in the TCGA cohort.

Cell type	Low risk	High risk	*p* value
Naive B cells	0.040267	0.02912	0.005183
Memory B cells	0.004514	0.001859	0.026328
Plasma cells	0.058277	0.03223	8.52*E*-06
CD8 T cells	0.108232	0.087539	0.006781
Naive CD4 T cells	0.002498	0.003162	0.638828
Resting CD4 memory T cells	0.072697	0.096681	0.000199
Activated CD4 memory T cells	0.051323	0.044211	0.100323
Follicular helper T cells	0.045584	0.029642	7.57*E*-08
Regulatory T cells (Tregs)	0.035	0.021083	2.45*E*-07
Gamma delta T cells	0.002973	0.001212	0.019656
Resting NK cells	0.024675	0.034967	0.000234
Activated NK cells	0.014214	0.014061	0.939139
Monocytes	0.002964	0.002761	0.78523
M0 macrophages	0.214181	0.256082	0.002617
M1 macrophages	0.087477	0.095573	0.146756
M2 macrophages	0.091557	0.109234	0.000378
Resting dendritic cells	0.046756	0.040619	0.204292
Activated dendritic cells	0.035284	0.034777	0.902059
Resting mast cells	0.027813	0.023826	0.160864
Activated mast cells	0.023828	0.03231	0.057819
Eosinophils	0.000594	0.000908	0.30239
Neutrophils	0.00929	0.008142	0.502245

## Data Availability

The gene expression profile data and clinical information of HNSCC patients in the GSE65858 dataset were obtained from the GEO database (https://www.ncbi.nlm.nih.gov/geo/). The gene expression profile data and survival information of patients (494 cases) with HNSCC in The Cancer Genome Atlas (TCGA) database were obtained from the University of California, Santa Cruz, Xena website (https://xenabrowser.net/datapages/).

## Supplementary Materials

Supplementary Materials Supplement Figure 1: the stability of this prognostic model was confirmed in HNSCC samples from TCGA. Supplement Table 1: number of genes corresponding to each module. Supplement Table 2: univariate and multivariate analyses of prognostic factors and overall survival of HNSCC patients in the TCGA cohort.
