# Supplementary material for: Identification of Immune-Related Prognostic Biomarkers Associated with HPV-Positive Head and Neck Squamous Cell Carcinoma
Source: J Immunol Res. 2021 Jan 8;2021:6661625. doi: 10.1155/2021/6661625 (PMC7810542; doi:10.1155/2021/6661625)
Supplement: Supplementary Materials — Supplement Figure 1: the stability of this prognostic model was confirmed in HNSCC samples from TCGA. Supplement Table 1: number of genes corresponding to each module. Supplement Table 2: univariate and multivariate analyses of prognostic factors and overall survival of HNSCC patients in the TCGA cohort. [file 6661625.f1.pdf]

Supplement Figure 1. The stability of this prognostic model was confirmed in HNSCC samples from TCGA.

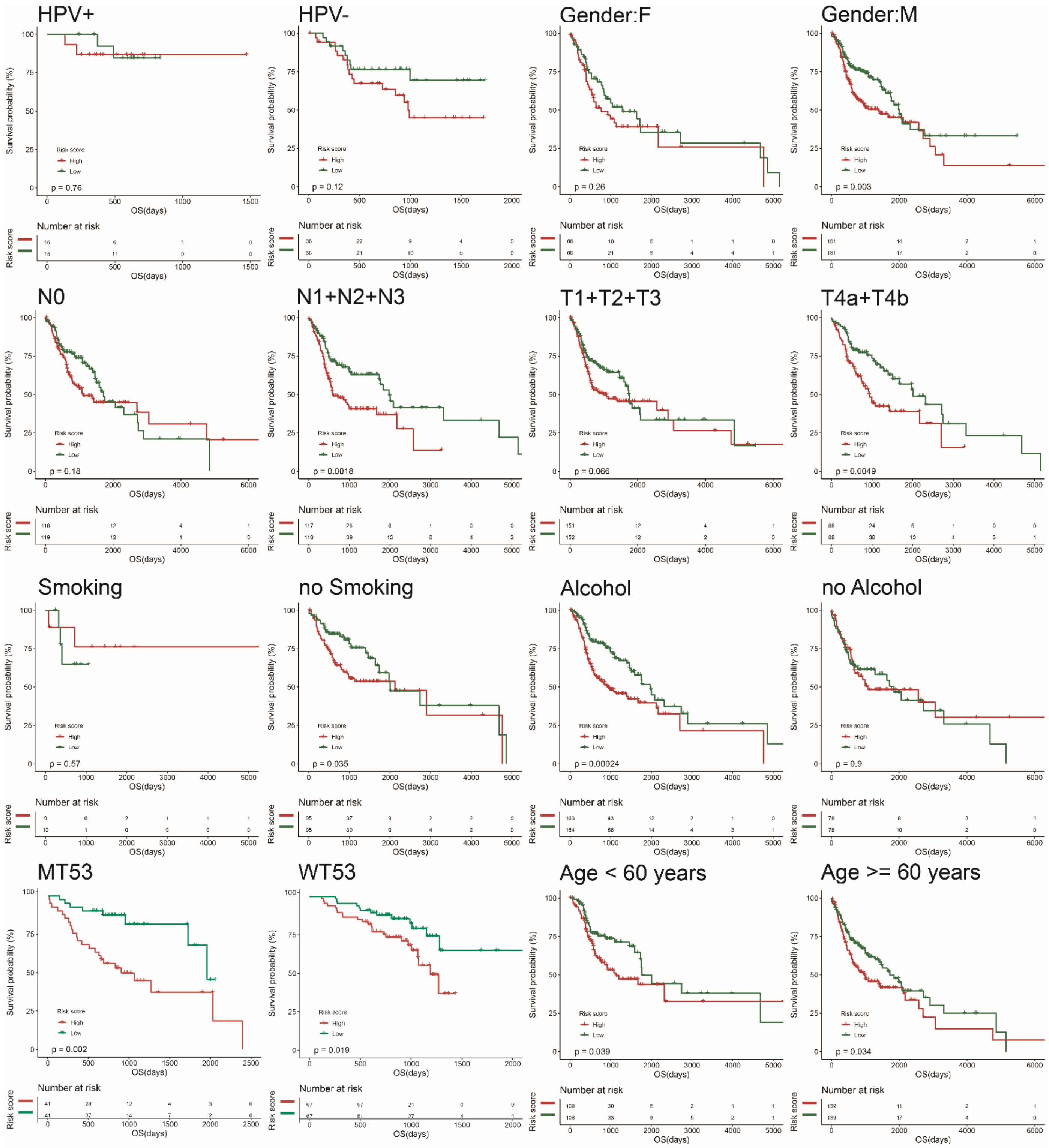

Supplement Table 1: Number of genes corresponding to each module

Module	No. of Genes
grey	219
blue	186
brown	128
turquoise	596
yellow	66

Supplement Table 2: Univariate and multivariate analyses of prognostic factors and overall survival of HNSCC patients in TCGA cohort.

	Univariate analysis				Multivariate analysis			
	HR	Low 95%CI	High 95%CI	P-value	HR	Low 95%CI	High 95%CI	P-value
N	1.14957002	0.988565	1.336797	0.070206	-	-	-	-
T	1.059054	0.919517	1.219766	0.426058	-	-	-	-
Age	1.02069555	1.007942	1.033611	0.001408	1.02	1	1.03	0.014061
Stage	1.08434272	0.931582	1.262153	0.295938	-	-	-	-
Gender	0.73316122	0.549764	0.977739	0.034581	0.85	0.63	1.15	0.289519
RiskScore	1.72596464	1.288336	2.31225	0.000254	1.63	1.21	2.2	0.001182
